# Case report and analysis: Behçet’s disease with lower extremity vein thrombosis and pseudoaneurysm

**DOI:** 10.3389/fimmu.2022.949356

**Published:** 2022-08-29

**Authors:** Han-Lu Wang, Jian-Hui Zhang, Yi-Cheng Wu, Jia-Li Lin, Yi Tang, Li-Sheng Liao, Jie-Wei Luo, Qing-Hua Yu, Zhu-Ting Fang

**Affiliations:** ^1^ Fujian Provincial Hospital, Shengli Clinical Medical College, Fujian Medical University, Fuzhou, China; ^2^ Electrocardiography Department of Xiamen Branch, Zhongshan Hospital, Fudan University, Xiamen, China; ^3^ Department of Interventional Radiology, Fujian Provincial Hospital, Fuzhou, China

**Keywords:** behçet’s disease, vascular involvement, deep vein thrombosis, aneurysm, diagnosis and treatment

## Abstract

**Background:**

Behçet’s disease (BD) is a unique autoimmune chronic systemic vasculitis that affects veins and arteries of all sizes. BD can lead to recurrent vascular events, especially venous thrombosis, with an incidence rate of 40%, or pseudoaneurysms formed under long-term inflammatory reaction or iatrogenic stimulation. BD-related risk factors promote endothelial dysfunction, platelet activation and overactivation of tissue factors leading to mural inflammatory thrombi. Thrombosis may be the first clinical manifestation of BD.

**Case presentation:**

A 32-year-old man complaining of progressive swelling and pain in the right lower extremity for 30 days was initially diagnosed with “venous thrombosis of the right lower extremity,” using color Doppler ultrasonography. Patient underwent inferior vena cava filter placement combined with deep vein angioplasty of the right lower extremity and catheter-directed urokinase thrombolysis. Postoperative oral anticoagulant therapy was administered. However, the patient was readmitted 20 days later for pulsatile pain in the right groin. Prior medical history included 4 years of repeated oral and perineal ulcers, and 2 months of blurred vision. Abdominal computed tomography angiography (CTA) revealed rupture of the right common iliac artery (CIA) and left internal iliac artery (IIA), complicated by a pseudoaneurysm. Based on the clinical manifestations and other auxiliary examination results, the patient was re-diagnosed with “BD combined with deep venous thrombosis of the right lower extremity and an iliac artery pseudoaneurysm.” Stent implantation was performed for iliac artery pseudoaneurysm after symptoms were controlled with timely immunosuppressive therapy. After endovascular treatment, the patient underwent continued immunosuppressive therapy and dynamic reexaminations of abdominal CTA, which revealed that a small amount of contrast agent at the stent in the right CIA continued to flow into the cavity of the pseudoaneurysm; in addition, the size of the pseudoaneurysm was gradually increasing. Therefore, the patient underwent a second stent implantation for iliac artery pseudoaneurysm, and the condition improved further.

**Conclusion:**

The importance of early diagnosis of BD should be recognized, and the choice of interventional and surgical procedures should be carefully evaluated, as this may trigger further damage to vascular access in BD patients with aneurysm.

## Introduction

Behçet’s disease (BD) is a chronic systemic vasculitis of unknown etiology, characterized by recurrent oral ulceration, genital ulceration, ocular lesions, cutaneous lesions, and involvement of multiple organs, including the vessels, heart, joints, lungs, nervous system, and gastrointestinal system (1). Although BD is prevalent worldwide, it is more common in the Middle East, the Mediterranean, and the Far East regions along the ancient Silk Road, resulting in its colloquial name as a “Silk Road disease.” The prevalence of BD is suspected 10.3/100000 worldwide and 14/100000 in China, while Turkey has the highest known prevalence globally with up to 420/100000 (2–4). The average age of BD onset is 35 years, and both sexes are equally affected, although young men experience more severe complications (5). More than one-third of patients with BD have vascular involvement. BD can affect the veins and arteries of any size, manifesting as thrombophlebitis, deep vein thrombosis, arterial occlusion, arterial stenosis, and aneurysms. The veins are usually more severely affected than the arteries. Venous thrombosis and aneurysms are the most common forms, with poor response to treatment, and a high risk of relapse, disability rate, and mortality (6). Clinically, vascular lesions of BD are usually confused with common vascular diseases, thus resulting in missed diagnosis or misdiagnosis, leading to serious consequences. Herein, we report a case of vascular involvement of BD, presenting with deep venous thrombosis of the right lower extremity and iliac artery pseudoaneurysm, and review the recent literature. We hope that this study will help enhance understanding and early identification of BD.

## Case description

**Figure 1:**
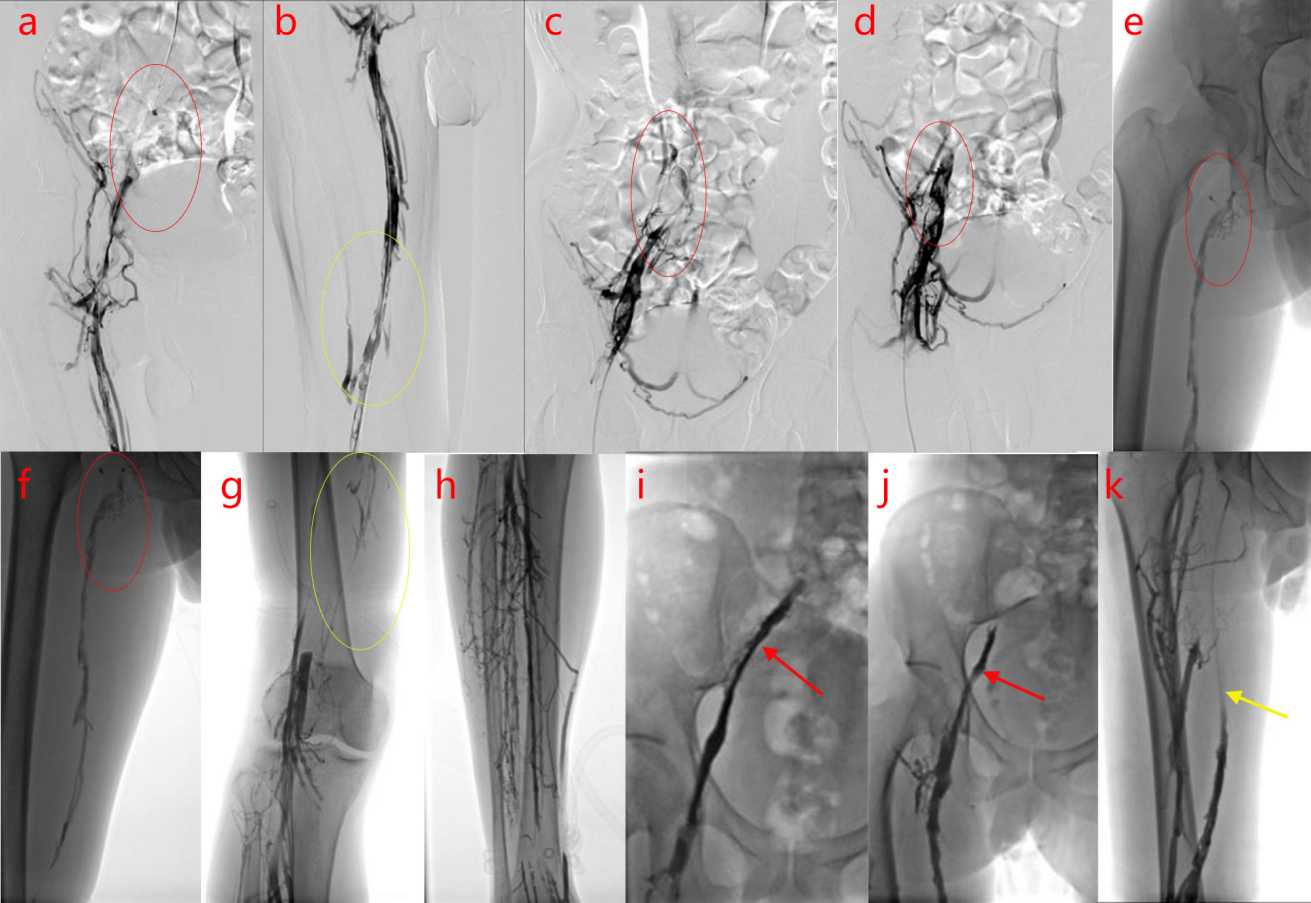
Digital subtraction angiography (DSA) of the right inferior vena cava examination. **(A–H)**.Before thrombolysis surgery, DSA of the patient reveals occlusion of the right common iliac vein (CIV) and right external iliac vein, with plenty of collateral vessels and extensive filling defects in the proximal and middle segments, exhibiting a stenosis degree of 50%–90% **(A–F)** femoral vein, **(G)** popliteal vein, **(H)** deep calf vein; the arrows and circles of different colors in the figures indicate the focus]. **(I–K)**. After thrombolysis surgery, DSA examination of the patient shows recanalization of the femoral vein (the arrows and circles of different colors in the figures indicate the focus).

A 32-year-old man was hospitalized for progressive swelling and pain in the right lower extremity for 30 days. Physical examination revealed variegated skin on the right lower extremity. His D-dimer level was 35.2 mg/L. Color Doppler ultrasonography revealed thrombosis in the right external iliac vein, common femoral vein, and superficial and deep femoral veins (up to the popliteal fossa). Patient underwent inferior vena cava filter placement, followed by deep vein angioplasty of the right lower extremity, and catheter-directed urokinase thrombolysis on November 02, 2016. Venography (**Figures 1A–H**) revealed occlusion of the right common iliac vein (CIV) and right external iliac vein with several collateral vessels, occlusion in the distal segments, and extensive filling defects in the proximal and middle segments of the right femoral vein, exhibiting a stenosis degree between 50%–90%. In addition, a 10 mm×8 cm balloon dilatation was performed in the right CIV, right external iliac vein, and right femoral vein, and a right CIV catheter was placed for thrombolysis. Urokinase was administered into the thrombus, with maintenance infusion of 20000 IU/h in the right CIV, and 30000 IU/h in the right foot dorsal vein. Five days later, deep venography of the right lower limb was performed, which revealed the following findings (**Figures 1I–K**): The signal in the right superficial femoral vein was unobstructed, the signal in the right deep femoral vein was unobstructed with several collateral vessels, the contrast agent returned to the right inferior abdominal vein and pelvic vein in collateral circulation; an obvious stenosis in the middle and distal segments of the right iliac vein and occlusion in the opening of the right iliac vein, and the contrast agent returned to the inferior vena cava through the right inferior abdominal vein and pelvic vein; unobstructed lumen and smooth wall of the inferior vena cava with a filter at a level slightly far from the opening of the renal vein. The inferior vena cava filter was subsequently removed. Post-operatively, warfarin dosage was adjusted to maintain an international normalized ratio of 2–3.

**Figure 2:**
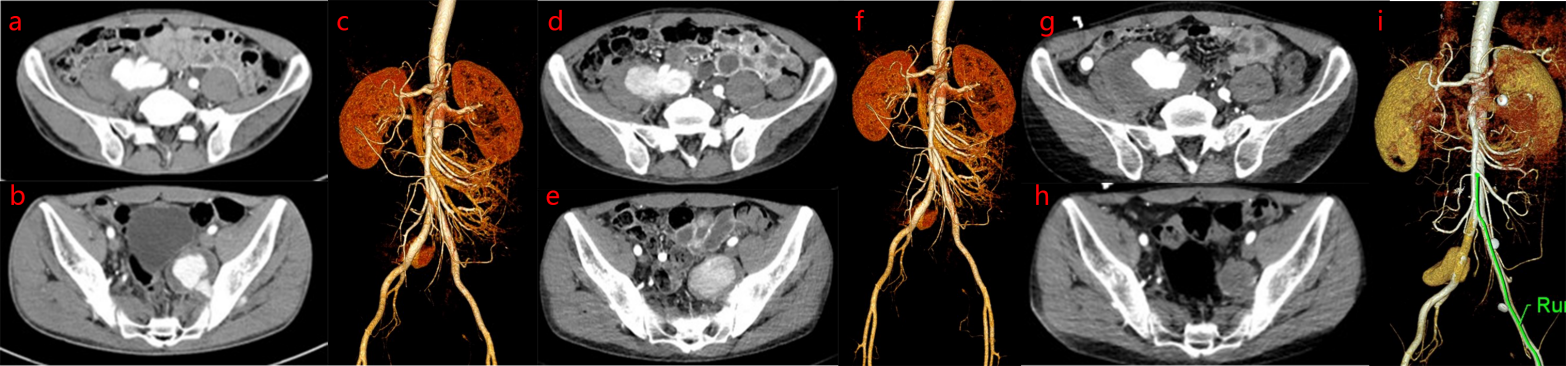
Abdominal computed tomography angiography (CTA) examination. Abdominal CTA images show dynamic progression of pseudoaneurysm based on drug intervention before bare stent implantation: the area of pseudoaneurysm of the right CIA is larger, while the area of the pseudoaneurysm of the left IIA is smaller. **(A-C)**. On admission abdominal CTA revealed rupture of the right common iliac artery (CIA) and left internal iliac artery (IIA), which was complicated by the formation of a pseudoaneurysm. **(D–F)**. After 1 month of drug therapy, abdominal CTA revealed that the area of the pseudoaneurysm of the right CIA was larger than that at the first examination, while the area of the pseudoaneurysm of the left IIA was similar to that at the first examination. **(G–I)**. After 10 months of drug therapy, abdominal CTA revealed that the area of pseudoaneurysm of the right CIA was larger than that at the second examination, while the area of pseudoaneurysm of the left IIA was smaller than that at the second examination.

Patient was discharged with complete resolution of leg edema. However, he was readmitted 20 days later for pulsatile pain in the right groin. A detailed investigation of his previous medical history revealed repeated oral and perineal ulcers for 4 years (>3 times/year), as well as 2 months with blurred vision. Physical examination revealed positive reactivity of the skin-to-needle prick (pathergy reaction). Laboratory tests showed that the erythrocyte sedimentation rate (ESR) was 25 mm/h, C-reactive protein (CRP) level was 34 mg/L, rheumatoid factor (RF) was negative, and antinuclear antibody (ANA), anti-double stranded deoxyribonucleic acid (anti-dsDNA), anti-Sm antibody, and anti-cardiolipin were all negative. Abdominal computed tomography angiography (CTA) showed the following findings: a cystic low-density shadow at the medial edge of the right psoas major muscle, with a range of 6.6 cm×5.6 cm, and contrast agent was observed to infiltrate into the cyst from the right common iliac artery (CIA) on enhanced scan. A cystic low-density shadow on the medial side of the left anterior sacroiliac muscle was discovered, with a size of 5.1 cm×4.3 cm. The contrast agent was observed to infiltrate the cyst from the left internal iliac artery (IIA) on enhanced scan, suggesting rupture of the right CIA and left IIA, complicated by the formation of a pseudoaneurysm (**Figures 2A–C**). Patient was diagnosed with BD in the active phase on the basis of clinical manifestations and auxiliary examination results. Methylprednisolone (40 mg/day) followed by infliximab injections (300 mg, 6 cycles) was administered as systemic treatment. Abdominal CTA was performed after 1 and 10 months of drug therapy, which showed the following findings: the area of the pseudoaneurysm of the right CIA increased from 6.8 cm×4.3 cm to 7.7 cm×6.3 cm, and the contrast agent still infiltrated the pseudoaneurysm from the right CIA on enhanced scan. However, the area of the pseudoaneurysm of the left IIA decreased from 5.5 cm×4.3 cm to 3.4 cm×3.0 cm, and no contrast agent was observed flowing gradually in the area of the left IIA pseudoaneurysm on enhanced scan (**Figures 2D–F,G–I**).

**Figure 3:**
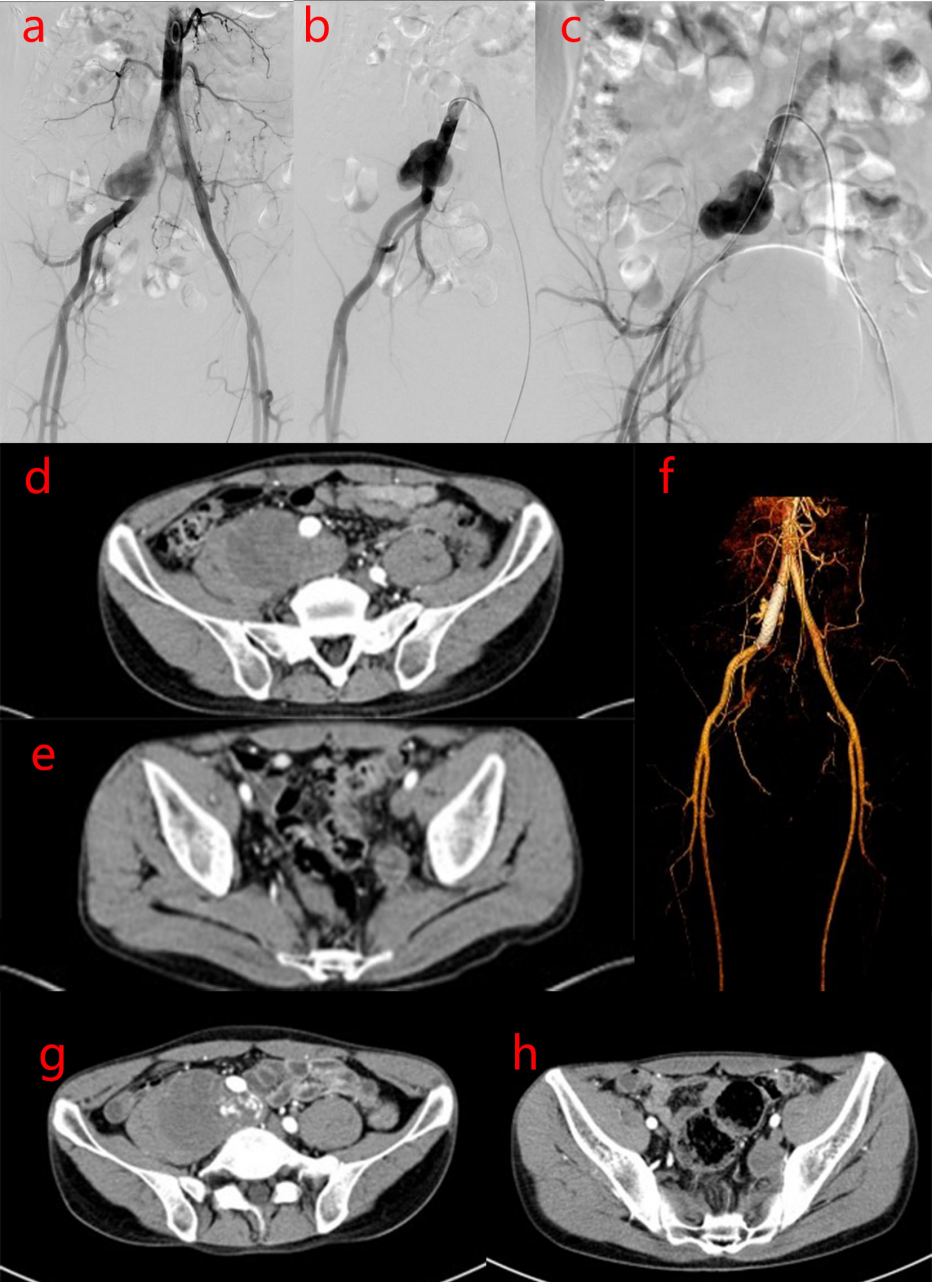
Imaging manifestations of pseudoaneurysm of the right CIA during bare stent implantation and after operation. **(A–C)**. After bare stent implantation, DSA of the right inferior vena cava showed the following findings: the signal within the stent was unobstructed in the right CIA; a small amount of contrast agent at the stent in the right CIA still flowed into the cavity of the pseudoaneurysm and the size of the pseudoaneurysm became small; there was no contrast agent in the area of the left IIA pseudoaneurysm. **(D–F)**. After 4 months of bare stent implantation, abdominal CTA showed that there is a small amount of contrast agent at the stent in the right CIA that continues to flow into the cavity of the pseudoaneurysm, leading to a slight increase in the size of the pseudoaneurysm. However, there was no contrast agent in the left IIA pseudoaneurysm area. **(G, H)**. After 11 months of bare stent implantation, abdominal CTA showed a small amount of contrast agent at the stent in the right CIA that continued to flow into the cavity of the pseudoaneurysm, and the size of the pseudoaneurysm was similar to that of **(D–F)**. However, there was no contrast agent in the left IIA pseudoaneurysm area.

**Figure 4:**
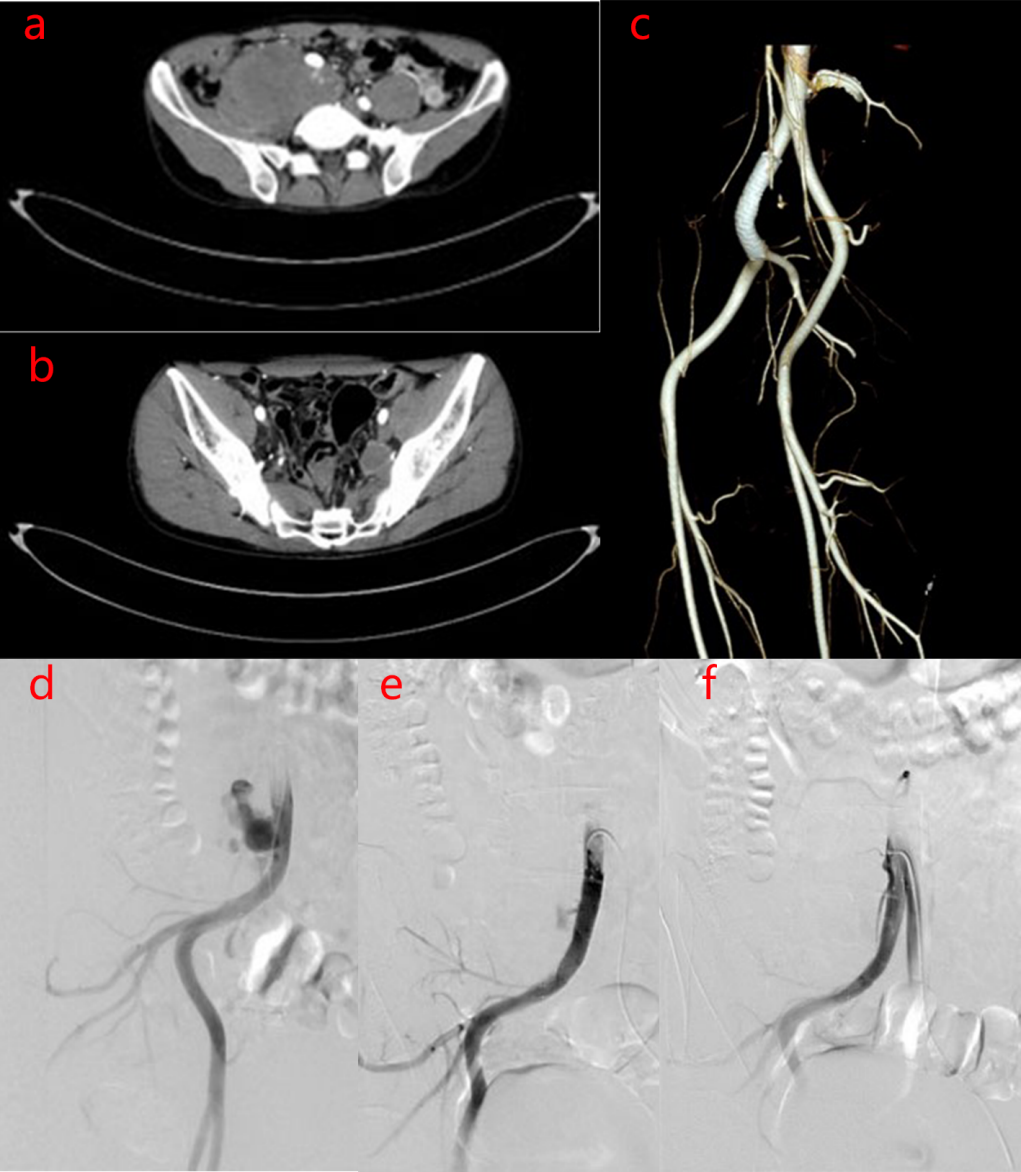
Imaging manifestations of pseudoaneurysm of the right CIA before and after covered stent implantation. **(A–C)**. After 27 months of bare stent implantation, abdominal CTA showed the following findings: The size of the pseudoaneurysm was obviously increased compared to that post-operation, because the contrast agent continuously flows into the cavity of the pseudoaneurysm. However, there was no contrast agent in the left IIA pseudoaneurysm area. **(D–F)**. After covered stent implantation, DSA of the right inferior vena cava showed the following findings: There was less contrast agent blowing into the cavity of the pseudoaneurysm of the right CIA and the size of the pseudoaneurysm was decreased.

After one year the patient was readmitted on November 14, 2017 for an increscent pseudoaneurysm of the right CIA. Physical examination revealed no positive findings. Laboratory testing showed an ESR of 2 mm/h and CRP levels <0.754 mg/L. As the patient’s condition was controlled with 8 mg/day methylprednisolone, he underwent stent implantation within the right CIA. Duplicate 10 mm×6 cm bare stent (Bard^®^ Luminexx™) implantation was performed in the right CIA, and postoperative angiography revealed contrast agent blowing into the pseudoaneurysm cavity (**Figures 3A–C**). The patient was administered aspirin (100 mg/day) and methylprednisolone (8 mg/day), and dynamic abdominal CTA reexaminations were performed post-operatively. Abdominal CTA was performed at 4, 11, 27 months postoperatively, which showed the following findings: the signal within the stent was unobstructed in the right CIA, while a small amount of contrast agent at the stent in the right CIA still flowed into the cavity of the pseudoaneurysm; the size of the pseudoaneurysm was gradually increasing compared with post-operation, up to 9.7 cm×7.0 cm. However, no contrast agent was observed in the area of the left IIA pseudoaneurysm (**Figures 3D–F, G, H**, **Figures 4A–C**). This suggested that the laceration of the right CIA did not completely heal, despite the immunosuppressive therapy and bare stent implantation. Therefore, the patient underwent stent implantation within the right CIA once again. Venography revealed a stent shadow in the right CIA and contrast agent spraying out of the stent, complicated by the formation of a pseudoaneurysm of the right CIA, sized approximately 4 cm×5 cm. A 10 mm×5 cm covered stent (Bard^®^ Luminexx™) implantation was performed in the right CIA, followed by 10 mm×8 cm balloon dilatation to avoid poor adherence to the covered stent. Post-operative angiography revealed that there was less contrast agent blowing into the cavity of the pseudoaneurysm (**Figures 4D–F**). The patient continued immunosuppressive therapy after discharge. The patient was followed up for two years after bare stent implantation, and the postoperative course was in stable condition. The DSA examination results at 2 years after operation were similar to the first DSA examination results postoperatively, which showed there was less contrast agent blowing into the cavity of the pseudoaneurysm of the right CIA.

## Discussion

BD is a chronic, recurrent, systemic vasculitis that shares some features with autoimmune and autoinflammatory disorders (7). The clinical manifestations of this condition are complex and diverse. The etiology and etiopathogenesis of BD remain unclear, and may be related to genetic and immunological factors, and microbial infection (8, 9). Genome-Wide Association Studies have revealed strong associations between genetic susceptibility of BD and several polymorphisms in genes such as *HLA-B51*, *ERAP1*, *IL10*, *IL23R*-*IL12RB2*, *STAT4 (* 10–12). The *HLA-B51* allele located within the major histocompatibility complex locus on chromosome 6p is the strongest genetic susceptibility factor for BD in areas along the ancient Silk Road (13). Microbial infection may be a triggering factor for BD. Four peptides derived from the Heat shock protein 65 (HSP65) sequence have been identified by T cell epitopes mapping, which stimulate specifically γδ T cells from BD patients. These peptides showed significant homology with corresponding peptides derived from human HSP and may induce uveitis development (14–16). HSP65 and herpes simplex virus (HSV)-1, *Streptococcus sanguis* might have a common denominator, while cross-reaction leads to immunoreaction in individuals with hereditary susceptibility (16). HSV1-infected Institute of Cancer Research (ICR) mice have similar clinical symptoms with BD patients, including oral aphthous ulcers, genital ulcers, ocular lesions, cutaneous lesions, gastrointestinal ulcers, and arthritis. These symptoms were relieved after valaciclovir treatment in mice (17, 18). A correlation might be also found between hypersensitivity tests against streptococcal antigens and the development of BD clinical manifestations. The peptides of Bes-1 gene and HSP65 derived from those uncommon serotypes of oral S. sanguinis were presumed to stimulate the production of pro-inflammatory Th1 type cytokines in PBMCs from BD patients (19). The composition of the salivary and intestinal microbiomes may contribute to BD pathogenesis. Imbalanced flora also plays a role in BD development by triggering immune system dysfunction (20, 21). Recently, increasing evidence has indicated that abnormal immune responses may be important in BD pathogenesis. T helper (Th)1, Th17 expansion, and regulatory T (Treg) cell number reduction are suspected to act as the basis of BD pathogenesis. Imamura observed by immunohistochemistry analysis that cells infiltrating the intestinal lesions of BD patients were mainly CD4+T lymphocytes; moreover, mRNA expressions of IFN-α, Txk (a Th1-specific transcription factor) and CCR5 (a Th1-chemokine receptor) were up-regulated in the intestinal lesions, suggesting that mainly lymphocytes infiltrating to the intestinal lesions in BD patients were Th1 cells (22). Ferrante et al. also have confirmed the correlation between intestinal lesions and Th1 response in BD patients. They detected mRNA expressions up-regulation of Th1-related cytokines, such as TNF-α, IFN-α, IL-12P35 and IL27 at intestinal mucosa. A significantly increased expression of Th1-related cytokines was also detected in peripheral blood of BD patients (23). Compared to healthy individuals, the number of Th17 cells is increased in the peripheral blood of BD patients, while the number of Treg cells decreases. Additionally, *in vitro* normal CD4+ T cells stimulated with serum from patients with active BD showed increased Th17 cell differentiation and decreased Treg cell differentiation compared to CD4+ T cells stimulated with serum from BD patients in remission (24, 25).

Vascular involvement in patients with BD is a decisive factor in disease prognosis. Vascular involvement is observed in 40% of BD patients and tends to occur in active male patients (26). Moreover, 75% of vascular events occur within 5 years of BD onset (27). Veins and arteries of all sizes are affected, although veins are usually more severely affected than arteries. Superficial thrombophlebitis and deep vein thrombosis (DVT) are the most common venous manifestations. Lower extremity deep venous thrombosis (LDVT) is the most common vascular lesion in patients with BD, accounting for 60–80% of all vascular lesions (28). LDVT causes intermittent claudication, characterized by multiple vein involvement, poor response to treatment, high relapse risk, and bed recanalization effects. Moreover, more than half of LDVT patients with BD may develop severe post-thrombotic syndrome (29). DVT can occur in atypical sites, including the inferior and superior vena cava, hepatic and portal veins, cerebral sinuses, and right ventricle (30). Venous thrombi in patients with BD tightly adhere to the inflammatory vascular wall, and are not easily detached. Arterial involvement in BD is considered a unique vascular feature (31), which mostly manifests as arterial occlusion, arterial stenosis, and aneurysms. Aneurysms, which are often located in the aorta, pulmonary artery, femoral artery, etc., are the most commonly observed manifestations combined with mural thrombosis, whereas coronary artery involvement is less commonly observed (32, 33). Prognosis of BD patients with arterial involvement is unfavorable. Combined with aneurysm rupture, especially pulmonary aneurysm rupture, significantly increased the risk of death in BD patients (34). Pathergy, arterial occlusion, arterial stenosis, and arterial thrombosis are risk factors for aneurysm formation (35). The incidence of pseudoaneurysms is higher than that of true aneurysms in BD patients, and pseudoaneurysms are more likely to rupture (36), the formation of which is related to traumatic artery operation (37). The relapse rate of vascular involvement was 23% at 2 years and 38.4% at 5 years (38). Various vascular manifestations may appear alone or coexist in the same patient (39).

Vascular involvement is a major contributor to disability and mortality rates of BD patients. Therefore, early diagnosis and treatment of vascular lesions are very important for prognosis of BD patients (40). Currently, diagnosis of BD depends on clinical manifestations, however there are no specific pathological or laboratory tests to help diagnose BD. Diagnosis can be based on the International Study Group (ISG) for BD (41). Non-invasive examinations such as color Doppler ultrasonography, computed tomography angiography, and magnetic resonance angiography are usually preferred for diagnosis of BD vascular lesions, because vascular invasive operations in BD patients may induce thrombi or pseudoaneurysms (42). This raises the following question, regarding our case: is the occurrence of a pseudoaneurysm of the right CIA directly related to thrombolysis surgery of the right lower limb? We conjecture thrombolysis surgery may activate inflammatory response of local blood vessels or placement and removal of intravascular filter may physically damage vessels leading to aneurysm formation. It might explain why venous and arterial involvement in this case occurred within months.

Potential treatments for BD vascular lesions include drug therapy and surgery. Drug therapy is the basis for all treatments. Once patients are diagnosed with BD, they should immediately receive glucocorticoids, immunosuppressants, and biological reagents to inhibit the inflammatory response, before undergoing necessary surgical intervention. DVT in BD is characterized by inflammatory thrombosis (39) and is not easily detached; therefore, the risk of pulmonary embolism in BD patients is low. There is no clear evidence of an increased risk of thrombosis. Considering the particularity of venous thrombosis mechanisms in BD, anticoagulant and/or thrombolytic treatment may only increase the relapse risk of thrombosis and the rupture risk of coexistent aneurysms (43–45). Combination treatment with anticoagulants and immunosuppressive agents reduces the relapse risk of DVT in patients with BD compared with anticoagulants alone (RR:0.17). Compared with immunosuppressive agents alone, treatment with anticoagulants and immunosuppressive agents did not significantly prevent DVT relapse (RR0.75) (46). Therefore, the European Alliance against Rheumatism recommends the use of glucocorticoids combined with immunosuppressants in BD patients with DVT, rather than anticoagulants, antiplatelet, and antifibrinolytic treatment (46). However, in BD patients with refractory venous thrombosis without pulmonary artery aneurysms, anticoagulants may be considered; otherwise, this type of treatment may increase the risk of post-thrombotic syndrome (47). Furthermore, for BD patients with DVT, placement and removal of intravascular filter should be avoided as far as possible, because it is potentially dangerous and can worsen thrombosis, lead to aneurysm formation, and other complications.

Aneurysms associated with BD often require surgical interventions. The indications and timing of surgery for aneurysms depend on the anatomical position and clinical manifestations, including whether the aneurysm is ruptured or close to rupture, and whether the disease is active or in remission (48). Generally, ruptured or imminently ruptured aneurysms require timely surgical intervention; however, the risk of post-operative complications, such as anastomotic pseudoaneurysm relapse, aneurysm rupture, thrombosis, and anastomotic infection, is high. Therefore, surgery should be carefully timed, except for emergencies. Surgery should be avoided in patients with active vasculitis, and immunosuppressive therapy should be preferred over surgery in such cases (49, 50).

For the management of peripheral arterial aneurysms, which are small and asymptomatic and have a low risk of rupture, cyclophosphamide and high-dose corticosteroids are recommended (51, 52). Anti-TNF-α agents, particularly infliximab, may be a good alternative in managing active refractory vasculo-BD (53, 54). For the patients, when pseudoaneurysms were found, because he could not tolerate the side effects of cyclophosphamide and corticosteroids pulses therapy, he initially taken reduced doses of corticosteroids orally alone, it was under corticosteroids treatment failure infliximab was used to inhibit inflammation. The above treatment result was that the area of the pseudoaneurysm of the left IIA decreased, while the area of the pseudoaneurysm of the right CIA increased. Which may be related to poor response to conventional doses of infliximab (5 mg/kg). In a Phase 3, multicenter, open-label, prospective single-arm trial, increasing infliximab dose to 10 mg/kg could improve clinical symptoms and CRP levels in the intestinal BD patients who responded poorly or were intolerant to conventional dose (5 mg/kg) (55). In this case, after the 1st stent failed under full immunosuppressive therapy with anti-TNF and steroids, use of cyclophosphamide or an increase in infliximab dose and/or frequency would have been an appropriate option to inhibit inflammation. And the fact that inflammatory parameters were normal does not preclude local focus inflammatory reactions of blood vessels, which is where we should reflect on.

Open surgery recommended for larger vascular lesions in BD may cause a high relapse risk of post-operative pseudoaneurysm, even aneurysm rupture, due to a large wound surface and multiple vascular anastomoses. Recently, intravascular interventional therapy, which causes less damage, fewer complications, and lower mortality, has been increasingly used for the treatment of aneurysms associated with BD (56). However, vascular stent implantation surgery has some anatomical limitations. For example, it is difficult to implant a stent in a hemangioma located in the vital branch, and thromboses or pseudoaneurysms are easily formed at the edge of the stent graft. The short-term efficacy of vascular stent implantation surgery is satisfactory, but long-term efficacy is uncertain. Hence, the choice of surgical intervention between endovascular and open surgery is based on the specific situation of the aneurysm and the experience of the surgeon. Considering the large artery incision at the puncture site of the covered stent and the high incidence rate of postoperative complications, bare stents were prioritized for implantation at a pseudoaneurysm of the right CIA in this patient in the first operation. However, dynamic re-examinations of postoperative abdominal CTA showed that the laceration of the right CIA could not completely heal, and the pseudoaneurysm was gradually enlarged. Therefore, a covered stent was chosen for the second operation. Furthermore, it is suspected that BD patients are prone to activate the coagulation process and/or inflammatory response to induce thrombosis on the new surface of the vessel wound after surgery because of vessel endothelium injury and dysfunction; therefore, it is crucial to prevent postoperative thrombosis. However, there is still no consensus regarding the use of anticoagulants in patients with post-operative BD. Conversely, it is recommended that post-operative BD patients continue to use glucocorticoids and immunosuppressants, although the duration of treatment is difficult to be determined. However, early discontinuation of immunosuppressive treatment increased the relapse rate of vascular BD (57).

## Conclusion

Owing to its insidious onset, patients with BD often choose clinical departments for medical treatment. Analysis of the initial symptoms, especially after only a single clinical presentation, often leads to misdiagnosis. When the visceral system is involved, it is difficult for clinicians to diagnose BD, which may delay treatment and lead to poor prognosis. In our case, diagnosis of the primary disease was missed at the first visit, and invasive surgery was therefore performed, even under the condition of persistent inflammation. Pseudoaneurysm formation was not definitely diagnosed until the second visit. It is difficult to ignore the relationship between pseudoaneurysm formation and incorrect thrombolysis surgery. Therefore, when a young man visits the hospital for DVT, arterial occlusion, or aneurysm with unidentified etiology, clinicians should be vigilant and consider the possibility of BD. Clinicians need to realize that immunosuppressive treatment is the most fundamental treatment, and open surgery or interventional surgery should be avoided in the active stage of BD.

## Data availability statement

The raw data supporting the conclusions of this article will be made available by the authors, without undue reservation.

## Ethics statement

The studies involving human participants were reviewed and approved by the Ethics Committee of Fujian Provincial Hospital, China. The patients/participants provided their written informed consent to participate in this study. Written informed consent was not obtained from the individual(s), nor the minor(s)’ legal guardian/next of kin, for the publication of any potentially identifiable images or data included in this article.

## Author contributions

H-LW, J-HZ, and Y-CW performed acquisition, analysis, and interpretation of clinical data. H-LW, J-HZ, J-LL, and Y-CW drafted the manuscript. YT and J-WL provided critical revision of the manuscript. J-WL, Q-HY, and Z-TF designed and supervised the study. All authors read and approved of the final manuscript.

## Funding

This work was supported by Fujian Province Natural Science Fund Project (2021J02053, 2020J011096, 2020J011064), the Special Research Foundation of Fujian Provincial Department of Finance (No. 2020-822, 2021-917), and the Fujian Province Medical Innovation Foundation (No. 2021CXB001).

## Acknowledgments

We thank the patient for cooperating with our investigation and acknowledge all participants for their valuable contributions to this article.

## Conflict of interest

The authors declare that the research was conducted in the absence of any commercial or financial relationships that could be construed as a potential conflict of interest.

## Publisher’s note

All claims expressed in this article are solely those of the authors and do not necessarily represent those of their affiliated organizations, or those of the publisher, the editors and the reviewers. Any product that may be evaluated in this article, or claim that may be made by its manufacturer, is not guaranteed or endorsed by the publisher.

## Abbreviations

ANA, antinuclear antibody; anti-dsDNA, anti-double stranded deoxyribonucleic acid; BD, Behçet’s disease; CIA, common iliac artery; CT, computed tomography; CRP, C-reactive protein; CTA, computed tomography angiography; DSA, digital subtraction angiography; DVT, deep vein thrombosis; IIA, internal iliac artery; MHC, major histocompatibility complex; PCT, procalcitonin; HSP65, Heat shock protein 65.
